# Disease-associated variants of Gap Junction Beta 2 protein (*GJB2*) in the deaf population of Southern Punjab of Pakistan

**DOI:** 10.1371/journal.pone.0259083

**Published:** 2021-10-25

**Authors:** Nabila Kausar, Asma Haque, Muhammad Shareef Masoud, Nazia Nahid, Usman Ali Ashfaq, Ali Muhammad Waryah, Rashid Bhatti, Muhammad Qasim

**Affiliations:** 1 Department of Bioinformatics and Biotechnology, Government College University, Faisalabad, Pakistan; 2 Department of Molecular Biology and Biochemistry, Shaheed Zulfiqar Ali Bhutto Medical University, Islamabad, Pakistan; 3 Molecular Biology and Genetics Department, Medical Research Center, Liaquat University of Medical and Health Sciences, Jamshoro, Pakistan; 4 National Centre of Excellence in Molecular Biology, University of the Punjab, Lahore, Pakistan; University of Iowa, UNITED STATES

## Abstract

Hearing impairment (HI) is a highly heterogeneous genetic disorder and is classified into nonsyndromic (without any other clinical manifestations) and syndromic (if combined with other clinical presentations) forms. Variations in GJB2 gene are the leading cause of autosomal recessive nonsyndromic hearing loss (ARNSHL) in several populations worldwide. This study was carried out to investigate the prevalence of GJB2 variations in severe-to-profound hearing impaired families of Southern Punjab of Pakistan. Ten families segregating ARNSHL were recruited from different areas of the region. Sanger sequencing of GJB2 coding region was carried out. In two out of ten families, NM_004004:c.*71G>A (p.(Trp24*)) and NM_004004:c.358_360del (p.(Glu120del)) homozygous variants were identified as the cause of hearing loss. Our study showed that GJB2-related hearing loss accounts for at least 20% of all cases with severe-to-profound hearing loss in the Southern Punjab population of Pakistan.

## Introduction

Deafness is a prevalent sensory disorder affecting 1 in 650 infants worldwide, with a prevalence of 3.5/1000 in teenagers and 2.7/1000 in children making it the most common hereditary sensory impairment [1].

It is a multifactorial disorder associated with environmental and genetic causes. About 50–60% of deafness is due to genetic factors. Clinically, deafness can be classified into nonsyndromic and syndromic deafness. Nonsyndromic deafness is isolated deafness with no other clinical manifestations, while syndromic deafness is associated with other metabolic or physiological conditions. Nonsyndromic deafness has a prevalence of 70% and is highly heterogeneous [2]. In case of nonsyndromic deafness, the most common inheritance pattern is autosomal recessive. Until now, 87 autosomal recessive nonsyndromic loci and 77 genes have been identified. Out of 87 DFNB loci and 77 genes, 45 loci and 31 genes were localized in Pakistani families (https://hereditaryhearingloss.org/recessive-loci). Consanguineous marriages are very common in Pakistan, making this population a valuable source for studying genetic disorders [3]. Moreover, it is observed that the rate of hearing loss in Pakistan is about 1.6/1000 live births, greater than the global prevalence i.e., 1 in 1000 live births [4]. It is also estimated that up to 50% of hearing loss is due to *GJB2* variants in prelingual deafness [5]. In Pakistan, more than 50% ARNSHL is associated with *GJB2* [6]. It is found that genetic alterations in *GJB2* are also the most primary cause of nonsyndromic hearing loss in South Asia [7]. Up to 38% of the *GJB2* frequency has been reported in north of Iran [8]. Several variations in the *GJB2* gene have been detected and reported; some are recurrent variations while others are not so prevalent. The variant spectrum diverges significantly among populations and demonstrates ethnic biases, e.g., c.35delG is common among caucasoids with 1 in 51 carrier rate [9] while carrier rate of c.235delC is 1–2% in the Japanese [10]. Carrier rate of 7.5% of c.167delT is reported in the Ashkenazi Jews [11] and carrier rate of 11.6% of p.Val37Ile in Taiwan [12]. The overall prevalence of *GJB2* variants in different populations worldwide is significantly high, thereby highlighting the clinical significance of this gene for genetic testing.

Southern Punjab is a loosely defined territory in Punjab Province of Pakistan that encompasses the civil divisions of Bahawalpur, Multan, and Dera Ghazi Khan. It accounts for around 52% of the province’s total land and 32% of its population. It has a population of more than 34,743,590 inhabitants. To the best of our knowledge, the prevalence of *GJB2* in hearing impaired population of Southern Punjab of Pakistan has not been studied. Therefore, the present study was conducted to determine the prevalence of *GJB2* gene in Southern Punjab of Pakistan. Sequencing of coding exon of *GJB2* revealed two disease-associated variants; NM_004004:c.*71G>A (p.(Trp24*)) in NKDF01 and NM_004004:c.358_360del (p.(Glu120del)) in NKDF08. This research will help in genetic counselling of these families to avoid carrier to carrier or carrier to affected marriages, which will result in a decrease in the deaf population in Southern Punjab. Moreover, *GJB2* variants profiling for the hearing impaired population of Southern Punjab of Pakistan will be a valuable resource for the development of molecular genetic screening tests in the future.

## Subjects and methods

### Cohort ascertainment

This research work was approved by the Institutional Research Ethics Committee (IREC) of Govt. College University Faisalabad, Pakistan. For this study, 10 punjabi severe to profound deaf families with consanguineous marriages were recruited from Dera Ghazi Khan and Rajanpur districts of South Punjab of Pakistan. Written consent forms were signed by all participants after receiving information about the study. A total of 37 deaf individuals and 72 normal siblings/parents were enrolled for *GJB2* sequencing.

### Phenotype characterization

Multiple family members, including elders, were interviewed to obtain medical history and rule out the environmental and syndromic deafness. A physical examination was also carried out to confirm signs and symptoms of night blindness, goiter and skin pigmentation for some of the affected participants. Two affected and one normal individual from each family were subjected to otoscopic examination and pure tone audiometry was performed at various threshold levels ranging from 250 Hz to 4 kHz (Table 1). Tandem gait and Romberg tests were performed to assess the vestibular function in two affected and one normal individuals of each family.

**Table 1:** Clinical manifestation in families subjected to GJB2 sequence analysis.

Families	Ethnicity	No. of affected	Onset of hearing loss	Severity of hearing loss
NKDF01	Punjabi	9	Congenital	Severe to profound
NKDF02	Punjabi	5	Congenital	Profound
NKDF03	Punjabi	3	Congenital	Profound
NKDF04	Punjabi	7	Congenital	Profound
NKDF05	Punjabi	4	Congenital	Profound
NKDF06	Punjabi	8	Congenital	Profound
NKDF07	Punjabi	3	Congenital	Severe to profound
NKDF08	Punjabi	4	Congenital	Severe to profound
NKDF09	Punjabi	4	Congenital	Profound
NKDF10	Punjabi	6	Congenital	Severe to profound

### Genetic analysis

#### Genomic DNA extraction

10 ml of blood was collected from each person that was properly labelled and stored in 50 ml of Sterilin^®^ polypropylene tubes containing 400 ul of 0.5M EDTA. The genomic DNA was isolated from all blood samples using the non-organic method [13]. Isolated genomic DNA’s quantity and quality was determined by gel electrophoresis and spectrophotometer (Bio-Rad, Hercules, CA).

#### Sanger sequencing

DNA of one affected from each pedigree was subjected to Sanger sequencing. Primers (5‘-TGTGCATTCGTCTTTTCCAG-3’ and 5-‘GGGAAATGCTAGCGACTGAG-3’) were designed, synthesized, and were subsequently used for amplification of the coding exon of *GJB2*. ExoSAp treatment was applied to the amplified products and sequencing was done using the Big Dye (Big Dye Terminator v3.1 Cycle Sequencing Biosystems^®^ Kit). Genetic Analyzer 3730 (Applied Biosystems Inc) was used to run the sequencing products and data was analyzed with Chromas software version 2.6.6 (Technelysium Pty Ltd).

## Results

Sequencing of *GJB2* results in the identification of two reported disease associated variants NM_004004:c.*71G>A (p.(Trp24*)) and NM_004004:c.358_360del (p.(Glu120del)) in NKDF01 and NKDF08 respectively.

### Family NKDF01

**Fig 1:**
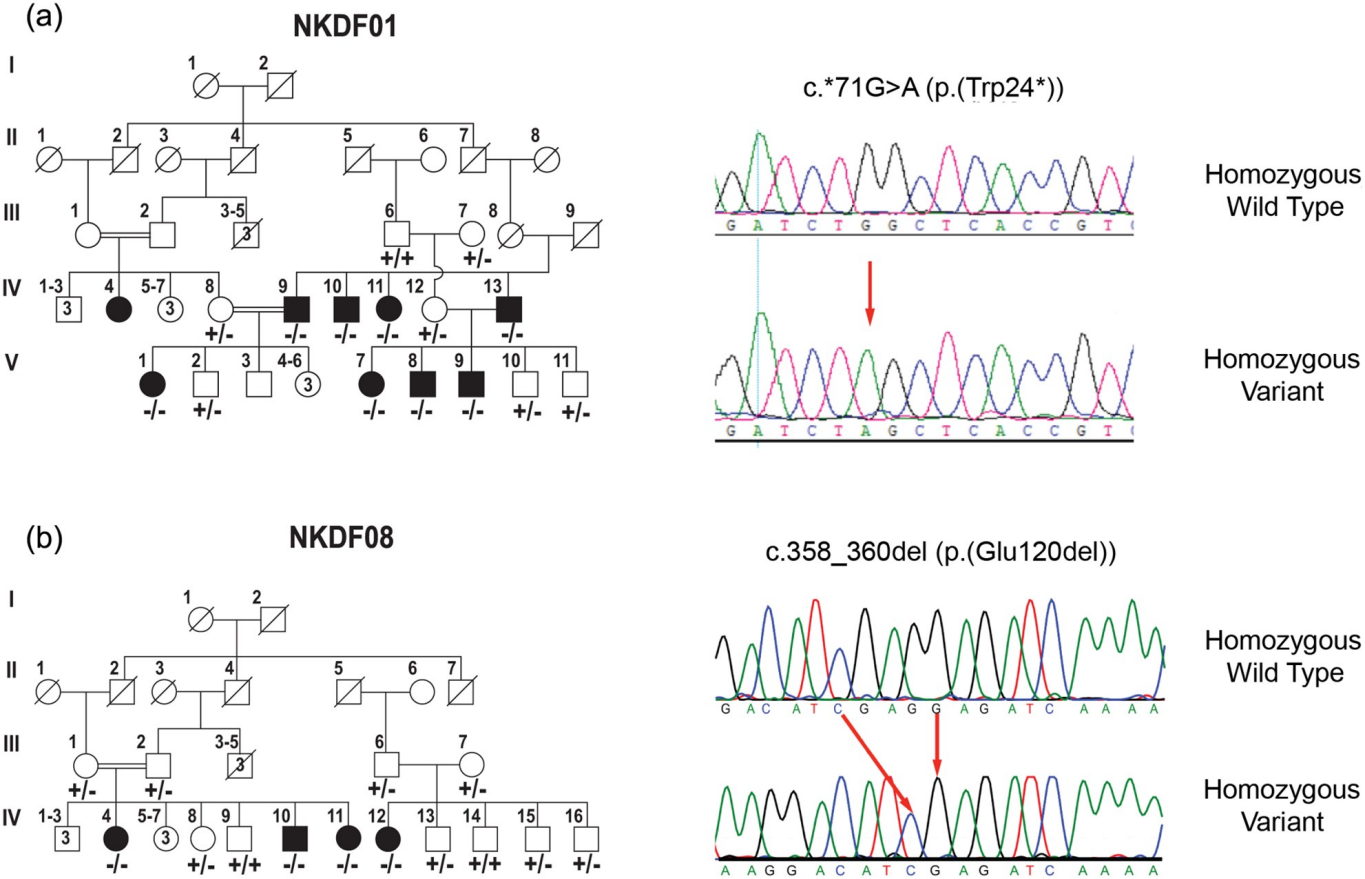
Family pedigrees of hearing impairment and their associated variants. (a) Pedigree of family NKDF01; Black squares and circles represent affected male and female participants, respectively. Consanguineous marriages are denoted by double horizontal lines. Under each symbol, the genotype for the *GJB2* variant is given. Sequencing chromatogram of family NKDF01 for individuals V-8 (Affected Son) and III-6 (Normal Father) is provided; the chromatogram shows a homozygous G>A change at 71 position of cDNA (c.*71G>A) in individual V-8. This change results in a stop codon at 24 amino acid position (p.(Trp24*)). The position of change is indicated by an arrow in the chromatogram. (b) Pedigree of family NKDF08 and sequencing chromatograms for individuals IV-9 (Normal Son) and IV-10 (Affected son). Chromatogram shows a homozygous c.358_360del change which results in p.(Glu120del) variant in individual IV-10. The position of change is indicated by arrows in the chromatogram.

This family was collected from Dera Ghazi Khan and had nine deaf individuals in four sibships. Eight affected individuals (IV:9, IV:10, IV:11, IV:13, V:1, V:7, V:8, V:9), three unaffected individuals (V:2, V:10, V:11) and their parents (III:7, IV:8, IV:12) were enrolled for this study (Fig 1a).

**Fig 2:**
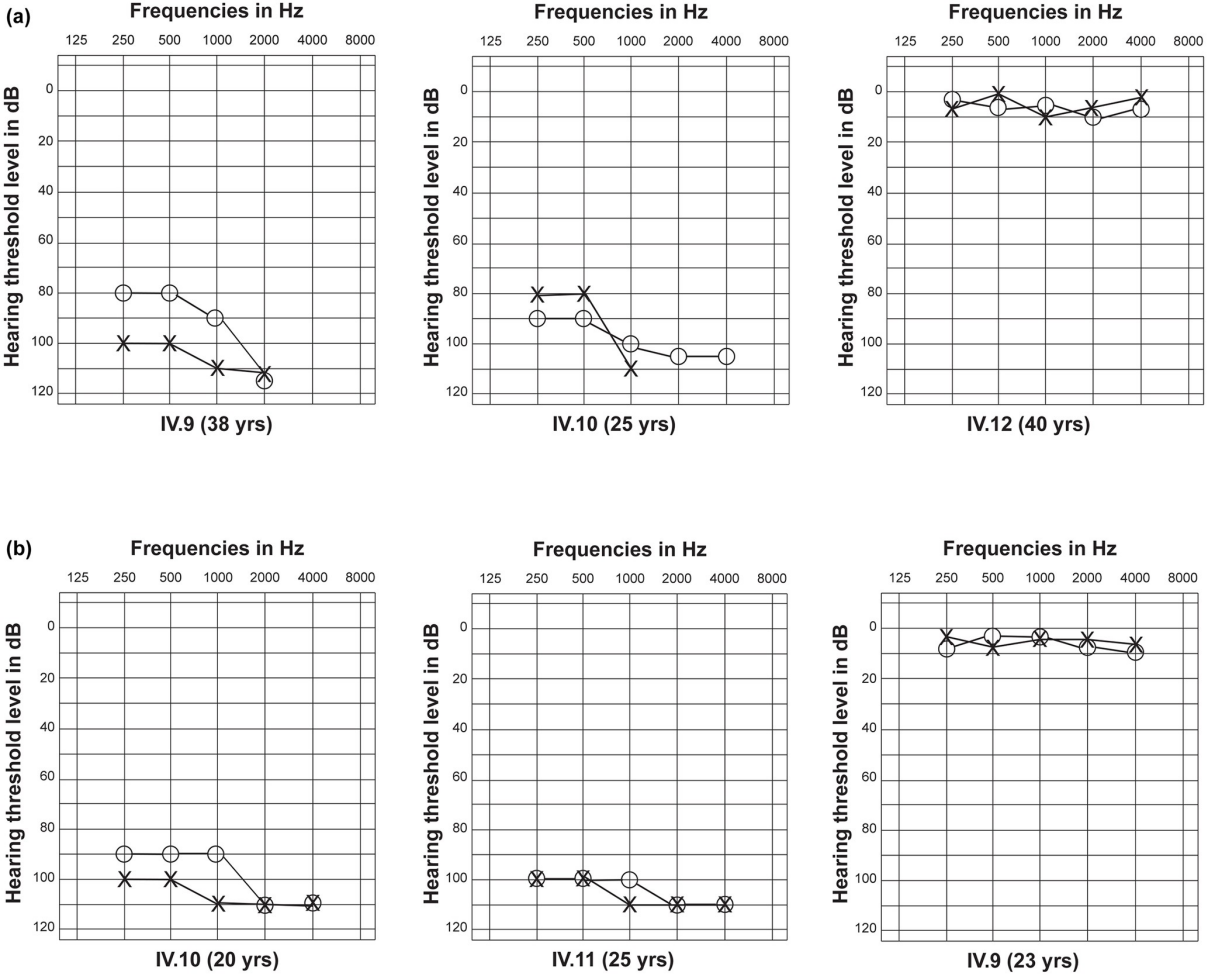
Audiograms of affected and normal individuals of NKDF01 and NKDF08. (a) Audiograms of two older affected individuals IV:9, IV:10 and a normal individual IV:12 of family NKDF01. Circles and crosses are representing the hearing thresholds for right and left ears respectively. Audiograms of both the affected individuals are depicting severe to profound hearing loss while the audiogram of normal individual (IV:12) is showing the normal threshold for hearing. (b) Audiograms of two older affected individuals IV:10, IV:11 and a normal individual IV:9 of family NKDF08. Audiograms are showing that both the affected individuals are having severe to profound hearing loss while the audiogram of normal individual (IV:9) is showing the normal threshold for hearing.

Physical examination did not exhibit the signs and symptoms of skin pigmentation, goiter and night blindness in any individual of this family. Furthermore, deafness in this family was not segregating with any other abnormality. No vestibular dysfunction was observed by tandem gait and rhomberg tests. Pure tone audiometry at 250, 500, 1000, 2000 and 4000 Hz for individuals IV:9 and IV:10 revealed severe to profound hearing loss (Fig 2a).

Sanger sequencing of *GJB2* gene identified a recurrent disease associated variant NM_004004:c.*71G>A (p.(Trp24*)) in deaf individuals of this family (Fig 1a). This nonsense variant was segregating in all the affected individuals of the family.

### Family NKDF08

This family was ascertained from Rajanpur and had four affected individuals in two sibships. In this family four affected individuals (IV:4, IV:10, IV:11, and IV:12), six normal individuals (IV:8, IV:9, IV:13, IV:14, IV:15 and IV:16) and their parents (III:1, III:2, III:6 and III:7) were ascertained (Fig 1b).

Hearing impairment was segregating without any other anomaly in the affected persons of this family. The signs and symptoms of goiter, skin pigmentation and night blindness in any individual of this family were not recognized. Moreover, tandem gait and romberg tests did not reveal the vestibular dysfunction. Pure tone audiometry demonstrated a severe to profound hearing loss in individuals IV:10 and IV:11 at 250, 500, 1000, 2000 and 4000 Hz (Fig 2b). Sanger sequencing revealed an in-frame deletion of three nucleotides i.e., c.358_360del (p.(Glu120del)) segregating with disease phenotype in this family (Fig 1b).

## Discussion

Variations in *GJB2* are the leading cause of hearing loss in Pakistani families [6]. Different studies had reported that c.*231G>A (p.Trp77*) and c.*71G>A (p.Trp24*) are the most prevalent variants of *GJB2* gene in Pakistani population [14–16]. Our results are also consistent with the previously published data which suggests that *GJB2* sequence variations are the primary contributor of deafness in Pakistani individuals. We did not perform the CNV analysis on the deaf families, however sanger sequencing revealed a c.*71G>A (p.(Trp24*)) change in NKDF01 family out of ten severe to profound hearing loss families. Hence, suggests a prevalence of 10% in this population. p.Trp24* frequency is also high in the Indian population reaching up to 95% [17–19]. Previously, it has been reported that both moderate to severe and profound hearing loss are associated with p.Trp24* in the Pakistani deaf population [14,20]. Hearing thresholds for affected individuals IV:9 and IV:10 of NKDF01 are also consistent with previous findings (Fig 2a).

Another *GJB2* variant c.358_360del (p.(Glu120del)) was observed in NKDF08 family, which exhibited a prevalence of 10% in Southern Punjab. Previously, this change was reported in only one Pakistani family [14]. However, it is the third and second most common change in Iran and Turkey respectively [21,22]. Moreover, phenotypic variability of mild and profound hearing loss was noticed in p.(Glu120del) individuals [23]. The common change in the Southern Punjab of Pakistan may be p.(Glu120del), but further sequencing from this region is required to confirm it. Because only two families were diagnosed with *GJB2* variants, the remaining families will be investigated further and subjected to exome sequencing to identify the causative genes.

## Conclusions

Our results suggest that the prevalence of *GJB2* related hearing loss in severe to profound deaf families is high in Southern Punjab, i.e., 20%. Sequencing of coding exon of *GJB2* revealed two disease-associated variants; NM_004004:c.*71G>A (p.(Trp24*)) in NKDF01 and NM_004004:c.358_360del (p.(Glu120del)) in NKDF08 out of ten families. As published earlier, p.Trp24* is the common change in the Pakistani families, while p.Glu120del has been identified in only one Pakistani family. Determination of reported variants along with other frequent variants will help in genetic counselling and family planning for these families, which will result in a decrease in the deaf population in Southern Punjab. Early detection of common *GJB2* variants in infants will enable us to adopt the multiple interventional strategies in time. Moreover, *GJB2* variants profiling for the hearing impaired population of Southern Punjab of Pakistan will be a valuable resource for the development of molecular genetic screening tests in the future.

## Supporting information

S1 Table Clinical manifestation in families subjected to *GJB2* sequence analysis. (DOCX)
