# Supplementary material for: Disease-associated variants of Gap Junction Beta 2 protein (GJB2) in the deaf population of Southern Punjab of Pakistan
Source: PLoS One. 2021 Oct 25;16(10):e0259083. doi: 10.1371/journal.pone.0259083 (PMC8544867; doi:10.1371/journal.pone.0259083)
Supplement: S1 Table — (DOCX) [file pone.0259083.s001.docx]

**Table S1:** Clinical manifestation in families subjected to *GJB2* sequence analysis

| **Families** | **Ethnicity** | **No. of affected** | **Onset of hearing loss** | **Severity of hearing loss** |
| --- | --- | --- | --- | --- |
| NKDF01 | Punjabi | 9 | Congenital | Severe to profound |
| NKDF02 | Punjabi | 5 | Congenital | Profound |
| NKDF03 | Punjabi | 3 | Congenital | Profound |
| NKDF04 | Punjabi | 7 | Congenital | Profound |
| NKDF05 | Punjabi | 4 | Congenital | Profound |
| NKDF06 | Punjabi | 8 | Congenital | Profound |
| NKDF07 | Punjabi | 3 | Congenital | Severe to profound |
| NKDF08 | Punjabi | 4 | Congenital | Severe to profound |
| NKDF09 | Punjabi | 4 | Congenital | Profound |
| NKDF10 | Punjabi | 6 | Congenital | Severe to profound |
